# Using a participatory approach to identify priorities to advance LHS implementation at an academic medical center

**DOI:** 10.1002/lrh2.10431

**Published:** 2024-05-26

**Authors:** Reid M. Eagleson, Madeline Gibson, Carletta Dobbins, Frederick Van Pelt, Allyson Hall, Larry Hearld, Andrea L. Cherrington, Jacob McMahon, Keith Tony Jones, Michael J. Mugavero

**Affiliations:** ^1^ Center for Outcomes and Effectiveness Research and Education University of Alabama at Birmingham Birmingham Alabama USA; ^2^ Department of Clinical Practice Transformation University of Alabama at Birmingham Birmingham Alabama USA; ^3^ Department of Health Services Administration University of Alabama at Birmingham Birmingham Alabama USA; ^4^ Department of Preventive Medicine University of Alabama at Birmingham Birmingham Alabama USA; ^5^ College of Science and Mathematics University of Auburn Auburn Alabama USA; ^6^ University of Alabama Health Services Foundation, UAB Medicine, and Clinical Affairs, University of Alabama at Birmingham Birmingham Alabama USA

**Keywords:** academic medical center, learning health system, learning health system implementation, participatory approach, precision problem solving

## Abstract

**Introduction:**

Like many other academic medical centers, the University of Alabama at Birmingham (UAB) aspires to adopt learning health system (LHS) principles and practices more fully. Applying LHS principles establishes a culture where clinical and operational practices constantly generate questions and leverage information technology (IT) and methodological expertise to facilitate systematic evaluation of care delivery, health outcomes, and the effects of improvement initiatives. Despite the potential benefits, differences in priorities, timelines, and expectations spanning an academic medical center's clinical care, administrative operations, and research arms create barriers to adopting and implementing an LHS.

**Methods:**

UAB's Center for Outcomes and Effectiveness Research and Education, in partnership with UAB Medicine's Department of Clinical Practice Transformation, applied part of the Precision Problem Solving methodology to advance the implementation of LHS principles at UAB.

**Results:**

Sixty‐two stakeholders across the UAB health system and academic schools noted 131 concerns regarding the development of an LHS at UAB. From the 131 items, eight major themes were identified, named, and prioritized through a series of focus groups. Of the eight major themes, “Creating a Structure for Aligned and Informed Prioritization” and “Right Data, Right Time, Improved Performance” ranked in the top three most important themes across all focus groups and became the critical priorities as UAB enhances its LHS. A task force comprised of diverse constituents from across UAB's academic medical center is taking first steps toward addressing these priority areas. Initial funding supports a prototype for enhanced health system data access and pilot projects conducted by researchers embedded in health system teams.

**Conclusion:**

We suggest that our experience conducting a deliberate process with broad engagement across both the health system and academic arms of the university may be informative to others seeking to advance LHS principles at academic health centers across a myriad of settings.

## 1 INTRODUCTION

Like many other academic medical centers, the University of Alabama at Birmingham (UAB) aspires to adopt and enhance implementation of learning health system (LHS) principles and practices. The Agency for Healthcare Research and Quality defines an LHS as “a health system in which internal data and experience are systematically integrated with external evidence, and that knowledge is put into practice.”^1^ Applying LHS principles establishes a culture where clinical and operational practices constantly generate questions and the requisite data, clinical documentation, clinical, financial, and operations data, and information technology (IT) infrastructure (“learning engine”) is leveraged by expert methodologists to facilitate evaluation of care delivery, health outcomes, and improvement initiatives.^2^ An LHS provides an integrated layer to consider study design, methods, and measures, and to conduct rigorous evaluation and dissemination of findings (“research engine”) allowing for the diffusion of lessons learned across units within the healthcare system, with the potential for disseminating generalizable knowledge to other health systems. Despite the potential benefits, differences in priorities, timelines, and expectations spanning the clinical care, administrative operations, and research arms of an academic medical center often prove formidable barriers to meaningful adoption and implementation of LHS principles.

The coronavirus disease of 2019 (COVID‐19) was a disruptive catalyst, advancing LHS discussions and principles at UAB.^3^ With a healthcare environment saturated in emerging epidemiological data, rapidly evolving prevention and treatment guidance, and a limited supply of hospital staff and resources, institutional agility and coordination was necessary to ensure appropriate care, accurate reporting, and continuous information sharing and uptake at the point of care. When faced with the urgency of COVID‐19, many historical barriers to implementing an LHS dissolved. UAB's academic and clinical arms responded to the dire need to develop and implement processes aligned with LHS principles more proactively and purposefully across the UAB enterprise. This clinical and academic alignment created the COVID Collaborative Outcomes Research Enterprise or COVID‐19 CORE, which engaged a collaborative working group to coordinate, collate, and facilitate the rigorous and expeditious completion of COVID‐19 health services, outcomes and population health studies conducted by interdisciplinary research teams addressing prescient questions germane to the individuals, communities and populations served by UAB.^3^ Aligned with LHS principles, the learnings from these studies informed care delivery and provided a foundation for grants to provide resources to support healthcare provider wellness among front‐line nurses, among others.^4^, ^5^

### 1.1 Research interest

Leveraging the advances made in response to the pandemic, leaders across the health system and academic arms of the university seized the opportunity to sustain LHS principles beyond COVID‐19. Using a novel approach to complex problem solving called Precision Problem Solving (PrecisionPS), a wide range of internal constituents participated in a process to identify and prioritize the barriers to sustaining LHS principles at UAB in a post‐pandemic era. Breaking down the false trichotomy that clinical care, healthcare operations, and research function independently, we describe the results of this process toward sustaining, accelerating, and expanding the implementation of LHS practices and principles at UAB beyond the learnings of the pandemic. UAB is a large urban university in downtown Birmingham, Alabama and includes Schools of Medicine, Nursing, Health Professions, and Public Health. The UAB Medicine enterprise consists of two‐hospital facilities with more than 1200 beds, and a multitude of specialty and primary care clinics as well as a freestanding emergency department and numerous satellite clinics statewide.

## 2 METHODS

**FIGURE 1:**
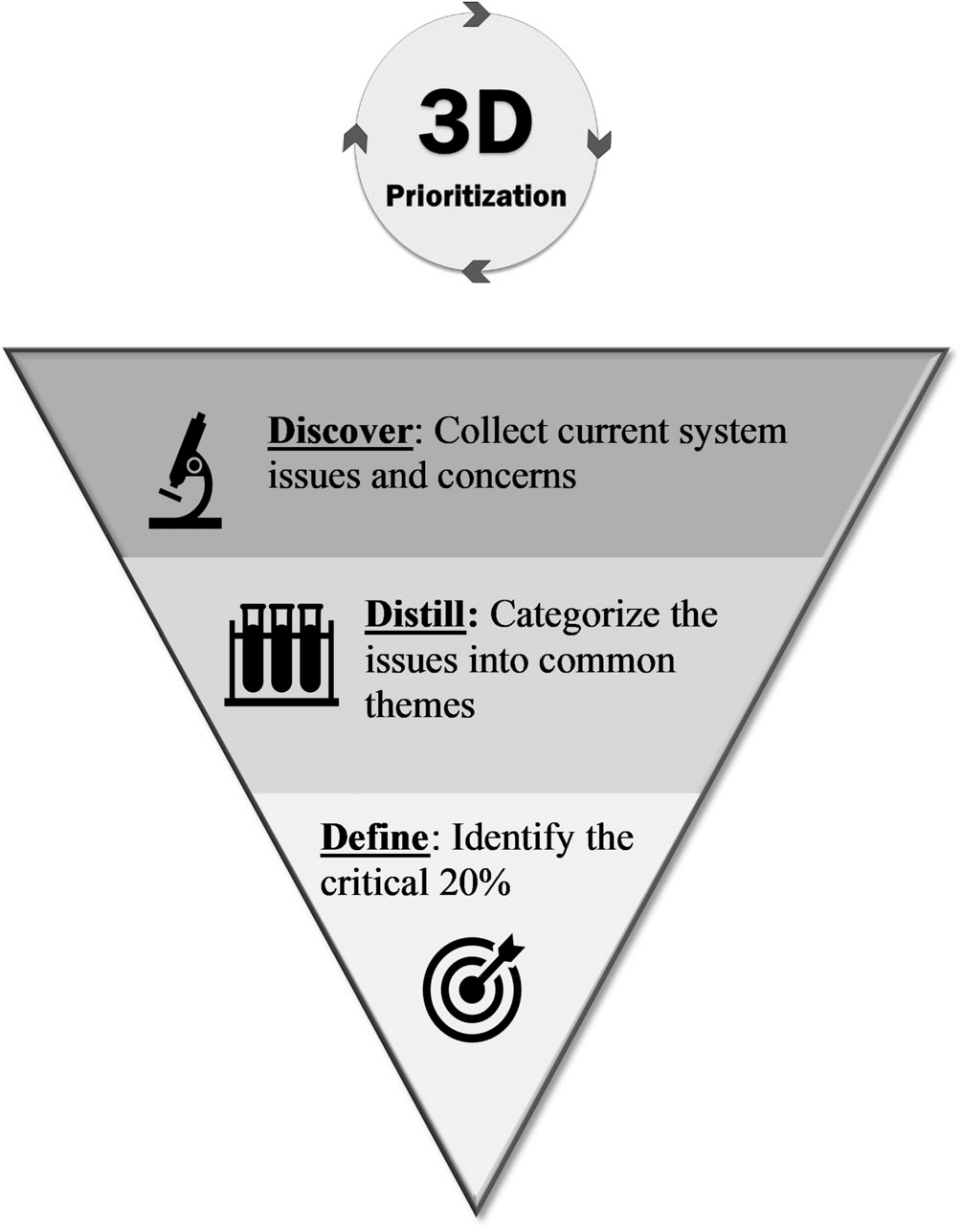
3D prioritization.

The UAB Medicine Department of Clinical Practice Transformation (CPT) developed the PrecisionPS methodology, encouraging a systems‐based participatory approach to complex problem‐solving and implementing high‐impact and sustainable solutions.^6^ PrecisionPS has three theoretical elements: systems theory, participatory management, and peer support. The application of PrecisionPS begins with a three‐step iterative process called 3D Prioritization (see Figure 1). In the first step, Discover, diverse constituents from the health system and academic arm of the university share system‐associated stressors and areas of friction impeding progress toward an objective. Discover concludes when participants have raised all their issues and concerns (i.e., saturation of themes). Participants, aided by facilitators, then categorize the previously raised issues into thematic groupings and collaboratively assign each grouping an action‐oriented name (the Distill step). Trello®, an electronic pinboard, provides real‐time visualization of the Discover and Distill steps. The final step of the 3D Prioritization process is Define, where stakeholders rank the themes according to priority or perceived impact. Inspired by the Pareto principle, where 20% of challenges affect 80% of outcomes, the Define step identifies the critical 20% that, if addressed, will yield high‐impact outcomes.^7^ The facilitator ensures an equitable and respectful conversation as participants agree on prioritization.

## 3 RESULTS

In February 2021, 3D Prioritization began with an interdisciplinary and interprofessional group of 62 leaders from across the UAB academic medical center enterprise who participated in four, sequential focus group sessions. Participants included leaders from across the ambulatory and inpatient clinical and operational teams in the health system, and academic investigators spanning all health professional schools at UAB. Health system operational participants consisted of a broad spectrum of members from ambulatory and inpatient settings, ranging from the Chief Compliance Officer, Chief Medical Officer, and Chief Medical Information Officer to directors of specific service lines such as respiratory and pharmacy. The team was careful to include other vice presidents and directors holding roles in patient experience, risk management, facilities, and informatics to help address as many aspects of creating an LHS as possible. Clinically, participants included registered nurses, physicians (e.g., primary care and subspecialists from ambulatory and inpatient settings), advanced practice providers. Academic and research participants held titles such as Dean, Senior Vice Dean for Research, and faculty of all ranks. During these sessions, the group identified 131 barriers to operationalizing an LHS at UAB in response to a guided question. These barriers ranged from needing to “bridge the gap between clinician‐facing data and data system management limitations and realities” to lacking “communication within the organizational framework, teams, and across campus.” Once no additional barriers and issues could be identified, Participants grouped the 131 items into eight major themes and 25 corresponding subtopics (see Table 1).

**TABLE 1:** LHS 3D themes and subtopics.

Right data, right time, improved performance	Roles, responsibilities, and rationale for data capture Access, analysis, and integration Data informed inquiry Data governance Data quality
Empowering integrated learning and innovation	Creating a culture of innovation Entrepreneurial strategy and investment in innovation Integrating innovation and shared governance
Creating a structure for aligned and informed prioritization	Inclusive engagement to inform prioritized Improvement decision‐making Synchronous informed sharing to facilitate aligned collaboration Clear communication of priorities Understanding what “LHS” is
Enhancing and integrating stakeholder engagement	Elevating the voice of the patient and care partners to the center of LHS decision‐making Empowering those who “do the job” to create the LHS culture Align UAB's academic structure worth LHS mission Design an aligned Strategy between leadership and communication infrastructure to enhance LHS outcomes
Cultivating engagement and collaboration with learning health systems beyond UAB	LHS Inquiry and Investigation LHS Distinction Beyond UAB
Realigning resources to support LHS priorities	Obtain funding and build infrastructure for research Prioritization of LHS initiatives with funding Aligning and prioritizing human resources to foster LHS initiative
Optimizing regulatory and administrative research processes	Optimizing regulatory and administrative research processes
Assimilating improvements into aligned LHS at UAB	Purposeful dissemination, implementation, and scale Aligned framework for improvement Integrating of best practice into clinical care

**FIGURE 2:**
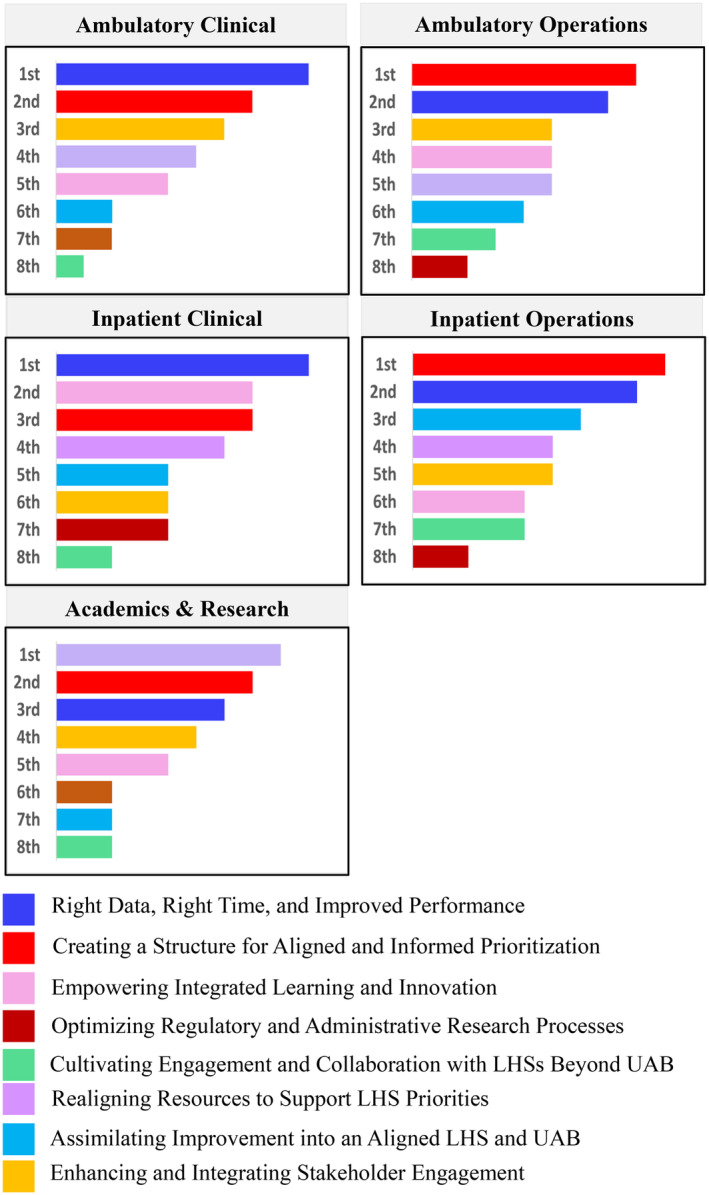
Ranking of themes in order of priority by individual stakeholder groups.

Following these sessions, the inaugural UAB Learning Health System Month then convened in March 2021 and included five weekly, public, one‐hour presentations via Zoom. During the last presentation, the facilitator showed the audience (*n* = 41) the eight themes previously identified and asked them to rank the themes in order of priority. Using Mentimeter®, audience members cast votes in real‐time. “Right Data, Right Time, Improved Performance”, and “Creating a Structure for Aligned and Informed Prioritization” tied as the most important, both receiving 29% of votes. “Realigning Resources to support LHS priorities” received 22% of votes. In August 2021, 96 health system and university representatives participated in a second Distill prioritization activity. These constituents were divided into five focus groups according to their primary affiliation: 19 from ambulatory clinical services, 30 from ambulatory operations services, 15 from inpatient operations services, 10 from inpatient clinical services, and 22 from the academic research arm of the university. Each group met with the CPT team virtually for 1 h and independently ranked the previously identified eight themes. Across all five groups, “Creating a Structure for Aligned and Informed Prioritization” and “Right Data, Right Time, Improved Performance” were ranked among the top three most important themes (see Figure 2), as they had been during the LHS month exercise. This solidified these two items as the critical 20%.

## 4 DISCUSSION

Informed by these findings, a 23‐member UAB LHS task force, including inpatient and ambulatory clinical and operations leaders from UAB Medicine, the UA Health Services Foundation, UAB central administration (e.g., IRB Director), the UAB Informatics Institute, and numerous academic research centers and investigators, took action. An interprofessional and interdisciplinary subgroup of the task force secured an intramural grant award in early 2022, allowing the task force to prototype a process to streamline and accelerate access to health system data and conduct three pilot projects with academic research methodologists embedded in clinical and operational teams. Taken together, these efforts aimed to address the “Right Data, Right Time, Improved Performance” theme. The process to request analysis‐ready health system datasets mirrored that of the COVID‐19 CORE, providing meaningful data to the three pilot projects and anyone across the UAB Enterprise needing access to health system data (35 datasets delivered between August 2021 and February 2024). The three pilot projects were interrelated and identified by UAB Medicine leaders. They aim to evaluate new and existing initiatives to reduce Emergency Department crowding. These evaluations are ongoing and will inform iterative clinical and operational enhancements. Findings will also be disseminated to additional internal UAB stakeholders and the larger scientific community by internal communication and peer reviewed abstracts and manuscripts, respectively.

In addition to carrying out these pilot projects, UAB's LHS has leveraged relationships and communication channels, developed via COVID CORE and the intramural grant period, to position ourselves as a resource and academic partner for the health system. This partnership intends to address the health system's priority needs, providing academic rigor and in‐depth evaluation complementary to a robust health system quality and safety infrastructure. Providing this added value to the health system is crucial to the sustainability of an LHS. Evolving priorities of the healthcare industry means that an effective LHS must be in tune with the needs of its administrative partners and provide synergistic support for new clinical and operational goals and objectives. While health system fiscal considerations have not been the initial focus of UAB's LHS, early efforts align with UAB Medicine's quality structure grounded in the Institute of Medicine's STEEP^8^ (Safe, Timely, Effective, Efficient, Equitable, Patient‐Centered) goals. For example, following the end of intramural funding, the UAB Department of Emergency Medicine is collaborating with the LHS team to systematically collect social determinants of health data and assess feasibility for future large‐scale implementation. The academic members of the LHS team are also exploring new transition of care collaborations, including grant submissions, based on findings from our intramurally‐funded pilot projects.

In the summer of 2023, UAB Medicine initiated a strategic planning process, having last done so in 2014. By participating in this strategic planning, UAB LHS task force members are addressing the second priority theme, “Creating a Structure for Aligned and Informed Prioritization.” While the results of these efforts remain to be seen, with a restructuring of the quality and safety units in the health system and strong support from health system leaders, we anticipate establishing a structure to support LHS alignment and informed prioritization. Such a structure will essentially need to be integrated within existing and planned units in the health system and interwoven with extant resources, infrastructure, and personnel from academic research Centers and Schools across campus. Along with internal structures, the LHS should maintain information exchange channels with external clinical, community organizations, and public health partners regarding healthcare trends and other shareable data. Lessons from contemporary programs, like those related to the COVID‐19 and HIV epidemics, provide exemplar strategies for LHSs to contribute to a larger learning health community across local or statewide geographies.^9^, ^10^, ^11^, ^12^

Notably, “Optimizing Regulatory and Administrative Research Processes” and “Cultivating Engagement and Collaboration with LHS's Beyond UAB” were consistently the lowest ranked themes across all five UAB focus groups. While Institutional Review Board (IRB) review and data use and sharing agreements are important, at a structural level, feedback from 3D Prioritization participants suggest these are less pressing relative to other themes. Nevertheless, as a first step to streamlining IRB application and approval for LHS projects and initiatives, a subgroup of the UAB LHS Task Force developed and launched a quality improvement self‐determination tool, informed by similar web‐based systems and guidance at peer universities.^13^, ^14^, ^15^, ^16^ Furthermore, through organic networking and professional relationships, academic research representatives of the UAB LHS task force engage with leaders at other academic medical centers and recently co‐sponsored a conference on the role of artificial intelligence in learning health systems, which is planned as an annual meeting.

UAB is not alone in seeking to advance LHS principles at a system level. Other large healthcare organizations and academic medical centers nationwide are developing their versions of an LHS. One such organization is Atrium Health, a large public, not‐for‐profit hospital system. At Atrium Health, a small group of research leaders collaborated with key healthcare administrators to create the Center for Outcomes Research and Evaluation. Key leaders like the Vice President of Analytics, Chief Strategy Officer, Chief Physician Executive, Chief Information Officer, and Chief Academic Officer were among those who championed the vision, culture, and institutional investment required to develop an LHS at Atrium Health.^17^ Academic medical centers like Vanderbilt University Medical Center and New York University Langone Health focus their LHS on generalizable knowledge generated by pragmatic comparative effectiveness trials and rapid‐cycle randomized testing.^18^, ^19^ Similar to UAB, these organizations took a participatory approach engaging a range of individuals spanning units in their academic health centers to developing an LHS. Many institutions also struggle with similar issues identified through our PPS process. Problems with obtaining timely and analyzable data, competing priorities, and overall health system culture are recognized barriers to LHS implementation.^20^, ^21^ The potential lack of alignment of priorities between the health system and academic enterprise is also a shared concern across all UAB focus groups and is a barrier at other LHS programs.^17^

## 5 CONCLUSION

We report our findings in the context of study limitations. As a single academic medical center in the Southeastern U.S., our experience and findings might not be generalizable to other academic medical centers or geographic regions. However, we emphasize the participatory process employed may hold broad value for other health systems seeking to advance LHS principles in their setting. Although we engaged many individuals in a myriad of roles (>200 participants) across the UAB enterprise and sought to create a “safe space,” it is possible that important items and/or themes were not shared during 3D Prioritization group sessions. It is also possible that participants that are more vocal steered the direction of focus groups during the Discover and Distill steps, although we note experienced facilitators actively sought input from all focus group participants. Moreover, using an individual rank order approach for the Define step ensured that each participant contributed equally to the final rank ordering of themes in the determination of the critical 20%.

In summary, we share findings of a single academic health center's systematic, stakeholder‐engaged process to determine actions and investments to advance implementation of LHS principles at UAB. Seizing upon the progress made during the COVID‐19 pandemic, 3D Prioritization elicited input from clinical, operational, and academic research leaders to identify two prevailing themes as the critical 20%: (1) enhancing timely access to actionable health system data, and (2) establishing a structure for aligned and informed prioritization of problems to be addressed within the health system. A task force comprised of diverse constituents from across UAB's academic medical center have taken initial steps toward addressing these priority areas. Intramural funding supported a prototype for enhanced health system data access and pilot projects conducted by researchers embedded in health system teams. Secondly, strategic planning in UAB Medicine provides potential for creating an aligned structure supporting LHS implementation, addressing this other critical barrier resoundingly identified by participants across the academic enterprise. While our results are forthcoming, we suggest that our experience in conducting a deliberate process with broad participant engagement holds potential not only to optimize LHS implementation, but also to enhance a sense of system‐wide culture and connectivity. This process may be informative and provide an example to others seeking to proactively advance LHS principles across a myriad of healthcare settings.

## FUNDING INFORMATION

This study received grant funding from the University of Alabama Health Services Foundation General Endowment Fund.

## CONFLICT OF INTEREST STATEMENT

The authors declared no potential conflicts of interest with respect to the research, authorship, and/or publication of this article.

## References

[1] About learning health systems . AHRQ. Published March 2019. Accessed March 6, 2023. https://www.ahrq.gov/learning-health-systems/about.html

[2] Anderson JL , Mugavero MJ , Ivankova NV , et al. Adapting an interdisciplinary learning health system framework for academic health centers: a scoping review. Acad Med. 2022;97(10):1564‐1572. doi:10.1097/ACM.0000000000004712 35675482

[3] Anderson JL , Reamey RA , Levitan EB , et al. The University of Alabama at Birmingham COVID‐19 Collaborative Outcomes Research Enterprise: developing an institutional learning health system in response to the global pandemic. Learning Health Syst. 2022;6(2):e10292. doi:10.1002/lrh2.10292 PMC8646452 34901441

[4] Polancich S , Patrician P , Miltner R , et al. Reducing hospital acquired pressure injury in a learning health center: making the case for quality. Learn Health Syst. 2023;7(3):e10355. doi:10.1002/lrh2.10355 37448459 PMC10336481

[5] Kennedy KC , Hearld KR , May B , et al. Inpatient telehealth and coronavirus disease 2019 outcomes: experiences in Alabama. Telemed Rep. 2021;2(1):148‐155. doi:10.1089/tmr.2021.0004 35720748 PMC8812284

[6] Dobbins C , Eagleson R , Martin H , et al. Precision Problem Solving: A Novel Methodology for Systems Transformation [Paper presentation]: European Academy of Management Annual Conference. 2023.

[7] Wright A , Bates DW . Distribution of problems, medications and lab results in electronic health records: the pareto principle at work. Appl Clin Inform. 2010;1(1):32‐37. doi:10.4338/ACI-2009-12-RA-0023 21991298 PMC3189502

[8] Six domains of Health Care Quality . AHRQ. Accessed April 22, 2024. https://www.ahrq.gov/talkingquality/measures/six-domains.html

[9] Using publicly available data to identify priority communities for a SARS‐CoV‐2 testing intervention in a southern U.S. state. Computer Weekly News. 2023:95.

[10] Bassler JR , Cagle I , Crear D , et al. Development and implementation of a distributed data network between an academic institution and state health departments to investigate variation in time to HIV viral suppression in the Deep South. BMC Public Health. 2023;23(1):937. doi:10.1186/s12889-023-15924-0 37226199 PMC10206341

[11] McCollum CG , Creger TN , Rana AI , et al. COVID community‐engaged testing in Alabama: reaching underserved rural populations through collaboration. Am J Public Health. 2022;112(10):1399‐1403. doi:10.2105/AJPH.2022.306985 35952331 PMC9480487

[12] Sohail M , Rastegar J , Long D , et al. Data for care (D4C) Alabama: clinic‐wide risk stratification with enhanced personal contacts for retention in HIV care via the Alabama quality management group. J Acquir Immune Defic Syndr. 2019;82(Suppl 3):S192‐S198. doi:10.1097/QAI.0000000000002205 31764254

[13] Decision tool: Does my project require IRB review? n.d. https://unmcredcap.unmc.edu/redcap/surveys/?s=XCC7FC4MPPEDR978

[14] Institutional Review Board . CHOP Research Institute. n.d. https://www.research.chop.edu/irb

[15] Quality Improvement Vs. research – do I NEED IRB …. n.d. https://research.vcu.edu/media/office-of-research-and-innovation/humanresearch/research_qi_guidance.pdf

[16] Surveys (Qualtrics) . UW–Madison Information Technology. 2024. https://it.wisc.edu/services/surveys-qualtrics/

[17] Taylor YJ , Kowalkowski M , Spencer MD , et al. Realizing a learning health system through process, rigor and culture change. Healthcare. 2021;8:100478. doi:10.1016/j.hjdsi.2020.100478 34175095

[18] Lindsell CJ , Gatto CL , Dear ML , et al. Learning from what we do, and doing what we learn: a learning health care system in action. Acad Med. 2021;96(9):1291‐1299. doi:10.1097/ACM.0000000000004021 33635834

[19] Horwitz LI , Kuznetsova M , Jones SA . Creating a learning health system through rapid cycle, randomized testing. N Engl J Med. 2019;381(12):1175‐1179. doi:10.1056/NEJMsb1900856 31532967

[20] Friedman C , Rubin J , Brown J , et al. Towards a science of learning systems: a research agenda for the high‐functioning learning health system. J Am Medic Inform Assoc. 2015;22(1):43‐50. doi:10.1136/amiajnl-2014002977 PMC4433378 25342177

[21] Morain SR , Kass NE , Grossmann C . What allows a health care system to become a learning health care system: results from interviews with health system leaders. Learn Health Syst. 2017;1(1):e10015. doi:10.1002/lrh2.10015 31245552 PMC6516720

